# Self-Assembled Fibrinogen Hydro- and Aerogels with Fibrin-like 3D Structures

**DOI:** 10.1021/acs.biomac.1c00489

**Published:** 2021-08-19

**Authors:** Dominik Hense, Anne Büngeler, Fabian Kollmann, Marcel Hanke, Alejandro Orive, Adrian Keller, Guido Grundmeier, Klaus Huber, Oliver I. Strube

**Affiliations:** † Institute for Chemical Engineering, University of Innsbruck, 6020 Innsbruck, Austria; ‡ Biobased and Bioinspired Materials, Paderborn University, 33098 Paderborn, Germany; § Physical Chemistry, Paderborn University, 33098 Paderborn, Germany; ∥ Technical and Macromolecular Chemistry, Paderborn University, 33098 Paderborn, Germany

## Abstract

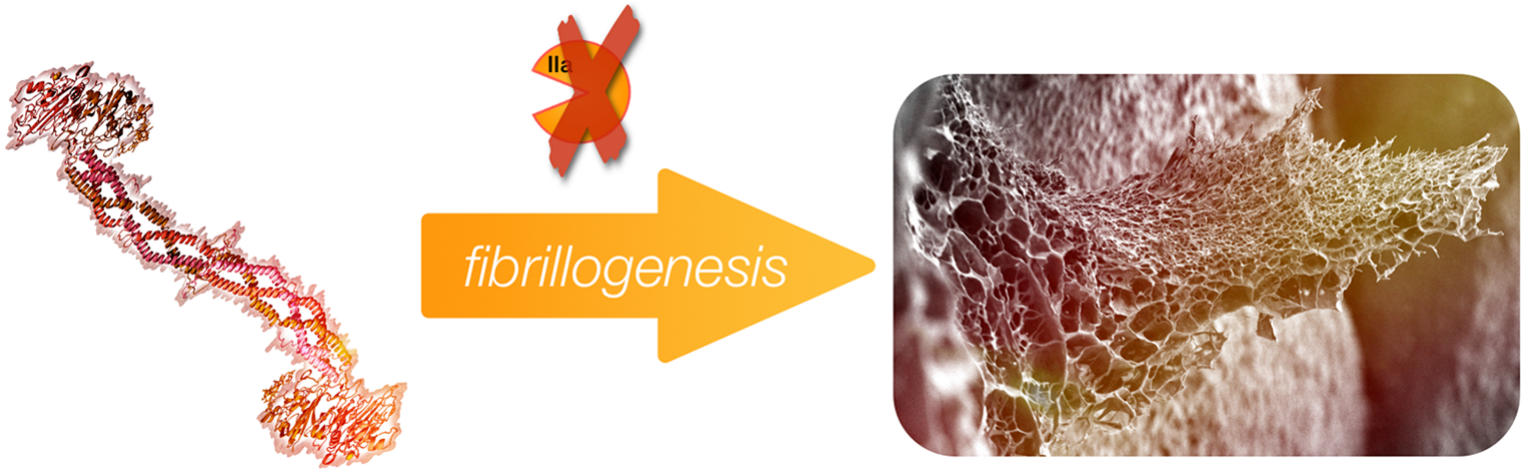

The natural blood protein fibrinogen is a highly potent precursor for the production of various biomaterials due to its supreme biocompatibility and cell interaction. To gain actual materials from fibrinogen, the protein needs to undergo fibrillogenesis, which is mostly triggered via enzymatic processing to fibrin, electrospinning, or drying processes. All of those techniques, however, strongly limit the available structures or the applicability of the material. To overcome the current issues of fibrin(ogen) as material, we herein present a highly feasible, quick, and inexpensive technique for self-assembly of fibrinogen in solution into defined, nanofibrous three-dimensional (3D) patterns. Upon interaction with specific anions in controlled environments, stable and flexible hydrogel-like structures are formed without any further processing. Moreover, the material can be converted into highly porous and elastic aerogels by lyophilization. Both of these material classes have never been described before from native fibrinogen. The observed phenomenon also represents the first enzyme-free process of fibrillogenesis from fibrinogen with significant yield in solution. The produced hydrogels and aerogels were investigated via electron microscopy, IR spectroscopy, and fluorescence spectroscopy, which also confirms the native state of the protein. Additionally, their mechanical properties were compared with actual fibrin and unstructured fibrinogen. The structural features show a striking analogy to actual fibrin, both as hydro- and aerogel. This renders the new material a highly promising alternative for fibrin in biomaterial applications. A much faster initiation of fiber formation, exclusion of possible thrombin residuals, and low-cost reagents are great advantages.

## 1 Introduction

Biological materials exhibit enormous potential in manifold fields due to their unique and outstanding properties. Usage of natural, biological materials has a long tradition and ranges from paints^1−3^ and wound dressings^4−6^ to specific nanoscale coatings by enzyme-mediated addressing.^7−9^

Among the natural materials of high interest is fibrin, the polymeric network responsible for wound closure. Mainly because of its biocompatibility and its fibrous, porous, and gel-like nature, it is considered a highly promising biomaterial for medical applications. Fibrin formation starts with the glycoprotein fibrinogen, which consists of two symmetric half-molecules linked by disulfide bridges.^10^ Each half-molecule contains three individual chains called α, β, and γ, and additionally the two fibrinopeptides A and B attached to the chains α and β. Enzymatic cleavage of A and B via thrombin triggers a spontaneous polymerization of fibrinogen^11^ to protofibrils that form branched networks upon lateral aggregation. After cross-linking with transglutaminases, like factor XIIIa, actual fibrin is formed.^10^

As a consequence of this synthesis, fibrin inherently possesses crucial disadvantages when considering it as a biomaterial. The most severe ones are slow formation kinetics, expensive components, and potentially harmful thrombin residues, which may cause thrombosis when released into the bloodstream.

To overcome these disadvantages, a broad spectrum of research is considering fibrin’s precursor fibrinogen as an interesting alternative. Instead of the biological cross-linking via enzymes, other approaches toward a stable network structure have to be developed. Especially salt-induced approaches are of high interest as it was, for example, investigated by Steven et al., who focused on fibrinogen aggregation triggered by metal ions.^12^ It remained however unclear whether a fibrous structure is gained that way.

Recently, a new approach to trigger fibrinogen self-assembly in thrombin-free media by lowering of ionic strength has been discovered.^13^ The aggregates obtained by that approach were studied in detail via time-resolved light scattering revealing compact spheres independent of the salt composition. In the context of drug delivery applications fibrinogen has also been manufactured into nanoparticles as carriers for 5-fluorouracil, a drug in cancer therapy.^14,15^ However, in both approaches the particles are not intended to be fibrous or even fibrin-like regarding their three-dimensional (3D) structure. Also, no sign of fibrillogenesis is reported.

One approach to actually create fibrinogen fibers in solution is described by Wei et al. Here, fibrillogenesis is induced by adding ethanol to dissolved fibrinogen. However, this process only yields extremely low amounts of fibers because of its strict limitations concerning the fibrinogen concentration. The optimum fibrinogen concentration for this approach is in the range of 5–50 mg/L. At higher concentrations, the process is inhibited and therefore not suitable for materials applications in its current form.^16^

On the other hand, several methods to create fibrinogen fibers in high amounts are reported, which however do not work in solution. One of these approaches focuses on electrospun fibrinogen mats for tissue engineering^17^ but requires enormously high protein concentrations of more than 80 mg/mL. Very recently, the potential to induce fibrillogenesis from fibrinogen by adding salt ions was described.^18,19^ The process occurs upon drying of the solutions on surfaces. Although fibrin-like fibrils are gained by this, gel-like, porous 3D structures are not accessible, and the necessity for a drying step limits the approach. Creation of solvent-born hydrogels is also not possible with this technique, again due to the necessary drying step.

In the present work, we describe a feasible, quick, and low-cost approach, which enables manufacturing of both nanofibrous hydrogels and aerogels from fibrinogen with very high structural and molecular resemblance of actual fibrin. In this novel process, gelation of fibrinogen is induced by specific anions at very distinct reaction conditions. Fibrillogenesis is observed immediately after addition of the trigger and after some time, hydrogels are gained in solution, which can also be lyophilized to gain highly porous and elastic aerogels. Moreover, the process requires only small amounts of fibrinogen and is performed directly in solution without expensive reactants or high energy demand. In the following, we outline the prerequisites and influencing parameters of the gelation and discuss the structural and molecular comparison to fibrin.

## 2 Experimental Section

### 2.1 Materials

Lyophilized fibrinogen from bovine plasma (≥99% protein of which ≥95% is clottable) was purchased from VWR. It contains traces of sodium citrate but was used without further purification. Fibrinogen was Thrombin (45 U/mg solid) was purchased from Sigma-Aldrich. The potassium phosphate buffer at pH 6.0 stems from Cayman Chemicals. All other salt stock solutions were prepared by weighing in the respective amount of salt and adjusting the pH to 7.0, if necessary. The anion concentration of all stock solutions is 500 mmol/L. 4-(2-Aminoethyl)benzenesulfonyl fluoride hydrochloride (AEBSF; purity ≥98%) was bought from Carl Roth. All experiments were carried out in pure water (HPLC grade, specific conductivity max. 1 μS/cm) by VWR. All other chemicals were of at least 98% purity and purchased from the usual suppliers.

### 2.2 Production of Hydrogels

Fibrinogen solutions with a concentration of 5 g/L were prepared by suspending the protein in 5 °C cold pure DI water and adjusting the pH to 7.0 with 0.1 M NaOH. Dissolution of the protein took 15 min. The mixture contains additionally <1 mmol/L sodium citrate stemming from the fibrinogen powder. However, these amounts were considered to be negligible.

The used salt stock solutions had a concentration of 500 mmol/L except the stock solution of pyromellitic acid, which had a concentration of 250 mmol/L due to solubility limitations. All stock solutions were adjusted to pH 7.0 with NaOH if not stated differently. To trigger fibrinogen self-assembly, 120 μL of the respective salt stock solution were added fast but without stirring. The final salt concentration in the reaction mixture was 15 mmol/L. For experiments using different salt concentrations, the volume of the added salt solution was adjusted accordingly. If not stated differently, the reaction mixture was stored for 4 h at 5 °C. To exclude unwanted changes in pH during pseudo-fibrin formation, the pH of analogously prepared samples was also monitored. These measurements proved that the pH remained constant after addition of the respective salt solution.

### 2.3 Production of Aerogels

The hydrogels were removed from residual solvent, frozen in liquid nitrogen, and lyophilized for 24 h. Fibrin hydrogels, fibrinogen in solution, and fibrinogen aggregated by reduction of ionic strength were lyophilized in the same manner.

### 2.4 Scanning Electron Microscopy (SEM)

Scanning electron microscopy was performed with a ZEISS “Neon 40”. Pictures of the samples were obtained by applying the SE2-detector or the InLens detector at an acceleration voltage of 2 kV.

### 2.5 Rheology

Rheological properties were monitored using an Anton Paar MCR 302. All samples were measured with cone/plate setup. To identify the linear viscoelastic region, an amplitude sweep was performed. Since all samples showed the desired behavior at a shear deformation of 1%, this value was used for the measurement of *G*′ and *G*″ over a frequency range from 0.01 to 10 Hz. For each measurement, a fresh sample was used.

Pseudo-fibrin hydrogels were prepared as described above. To trigger fibrillogenesis, a sodium phosphate buffer at pH 7 was used. Fibrin references were prepared in two different ways. One of them was prepared analogously to the pseudo-fibrin sample, i.e., 5 g/L were dissolved in 5 °C cold ultrapure water at pH 7. Clotting was induced by adding 5 U/mL thrombin. Since these conditions do not resemble physiological conditions (under which fibrin is usually prepared), a second sample was prepared in phosphate-buffered saline (PBS) at room temperature. Again, clotting was induced by addition of 5 U/mL thrombin. In addition, two reference samples containing only fibrinogen were prepared analogously to the two fibrin samples but without addition of thrombin. All five samples were stored for 4 h at the respective temperature at which they were prepared.

### 2.6 ThT Fluorescence and Attenuated Total Reflection Infrared (ATR-IR) Spectroscopy

Fluorescence spectra were obtained using a JASCO FP-8200 fluorescence spectrometer. At an excitation wavelength of 450 nm, the fluorescence was measured in the range of 460–700 nm in steps of 1 nm. The scan rate was 500 nm/min and the final spectra are the average of three measurements each. Fluorescence measurements were done as follows: 667 μL of a freshly prepared fibrinogen solution (7.5 g/L) was mixed with 303 μL of a thioflavin T (ThT) solution in ultrapure water. The final ThT concentration was 20 μmol/L in all cases. With this mixture, a reference spectrum was measured. Afterward, 30 μL of the thrombin solution or a potassium phosphate buffer with pH 6.0 was added to obtain fibrin or pseudo-fibrin, respectively. The final thrombin concentration was 5 U/mL, and the final phosphate concentration was 15 mmol/L.

### 2.7 ATR-IR Spectroscopy

Attenuated total reflection infrared (ATR-IR) measurements were done with a BRUKER Vertex 70 spectrometer. The absorbance was measured in the range 1250–2000 cm^–1^. From both reaction samples prepared for the ThT binding assay, 300 μL were removed and used for ATR measurements. The equilibration time on the freshly prepared ATR crystal was 30 min in all cases. Additionally, one reference ATR spectrum of a pure 5 g/L fibrinogen solution was measured.

### 2.8 Light Scattering Investigations

Dynamic light scattering (DLS) measurements at an angle of 90° were conducted on a Zeta Nano-ZS from Malvern Instruments. Additional time-resolved combined dynamic and static light scattering (DLS/SLS) were carried out with an ALV/CGS-3/MD-8 multidetection system from ALV-Laservertriebsgesellschaft (Germany, Langen). A He–Ne laser was used as a light source, with a wavelength of λ = 632.8 nm and a power of 35 mW. The cell housing with the toluene bath was equilibrated at 25 °C with an external thermostat throughout all measurements. The instrument includes an array of eight detectors with an angle of 8° between two neighboring detectors. The whole system covers an angular range of 30° ≤ θ ≤ 86°.

Molar mass values *M*_w_ were established via extrapolation of angular-dependent scattering intensities to zero scattering angle. The resulting values are apparent values and signal the scattering power of the particles as they increase with increasing particle mass. Size distributions are represented as intensity weighted distributions of hydrodynamic radii *r* based on a CONTIN analysis of the electric-field-time correlation functions.^20^

For each combined DLS/SLS measurement, a fibrinogen solution with a concentration of 0.2 g/L was freshly prepared by dissolving the protein in 20 °C warm DI water for 10 min. Sodium phosphate buffer stock solutions with concentrations of 10, 30, and 100 mmol/L were prepared. All stock solutions had a pH of 6.0.

The fibrinogen solution (1 mL) was filtered into the cuvette using 450 nm PES-membrane filters. To define the initial values of *R*_g_ and *M*_w_, for each sample, a DLS/SLS measurement was performed prior to phosphate addition. Afterward, to achieve final phosphate concentrations of 5, 15, and 50 mmol/L, 1 mL of the respective stock solution was filtered into the cuvette so that the final fibrinogen concentration was 0.1 g/L in all cases.

## 3 Results and Discussion

### 3.1 Phosphate-Induced Formation of Fibrinogen Gels

To produce fibrin-based materials *in vitro*, fibrinogen is usually dissolved in saline phosphate buffers (PBS buffer), which closely resemble *in vivo* conditions and contain high amounts of chlorides, more precisely 137 mmol/L NaCl. This procedure is certainly comprehensible as fibrinogen is not completely dissolved on the molecular level at lower NaCl concentrations, as has just recently been outlined via time-resolved light scattering.^13^

However, routine application of said conditions has apparently obstructed the recognition of a rather remarkable effect, which enables a novel, rapid, and highly feasible initiation of fibrillogenesis without enzymatic action. If indeed, a non-saline phosphate buffer (Na_2_HPO_4_/NaH_2_PO_4_) is utilized for dissolution of fibrinogen, an unexpected change of behavior can be observed. Though the protein still dissolves initially, a clouding effect is observed shortly after. This alone is curious because in this experiment, a medium, which acts as a solvent in the first case, suddenly turns into a nonsolvent after some minutes. This can only be rationally explained if two competing effects are at work. One effect is certainly enhanced dissolution because of increased ionic strength. The other one would have to be a specific interaction of the utilized salt(s), in this case phosphates, with the protein.

The rationale of such a specific interaction is supported by the fact that the observed aggregation is profoundly different from a conventional fibrinogen precipitation. Usually, amorphous protein would quickly deposit at the bottom of the reaction vessel after aggregation.^13^ In the case of phosphate-induced aggregation however, the cloudy structure is quite stable for about 1 h. After that, the material densifies and finally evolves into a floating, medusa-shaped, and mechanically semistable hydrogel. This observation is presented in Video 1 in the Supporting Information.

**Figure 1:**
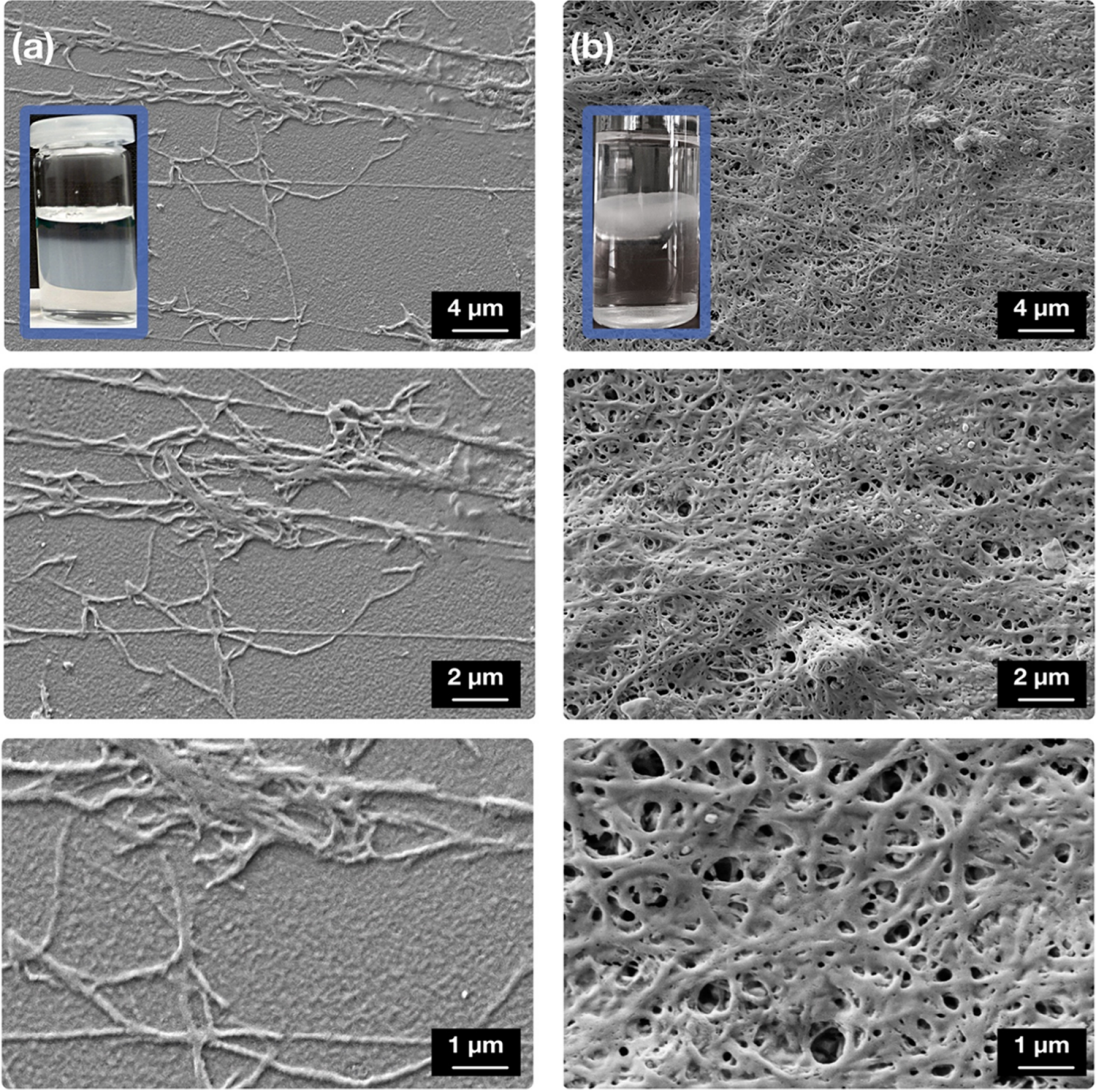
Stages of pseudo-fibrin evolution. (a) Instant “cloud” formation after addition of saline-free phosphate buffer; (b) densification to a medusa-shaped hydrogel structure after 5 h.

Even more alerting, the microscopic structure of the protein is highly fibrous, even in the initial stage while the final hydrogel-like material is an interconnected, fibrous network, as can be clearly seen from respective SEM pictures in Figure 1. Nonspecific aggregation of fibrinogen in contrast yields only amorphous material without any distinctive features. Moreover, the observed fibrous networks exhibit remarkable similarity to the well-known network structure of actual fibrin. Also the experimental observations, though quicker, also resemble fibrin formation via enzymatic action, but no enzyme should be involved at all. Thus, a control experiment in the presence of the serine protease inhibitor 4-(2-aminoethyl)benzenesulfonyl fluoride hydrochloride (AEBSF) was performed to rule out thrombin contamination of the biological base material as potential aggregation trigger. The result of these control experiments is however identical; a fibrous, hydrogel-like material is formed. Because of its high similarity to fibrin in behavior and microstructure, the new material will be referred to as pseudo-fibrin from now on.

**Figure 2:**
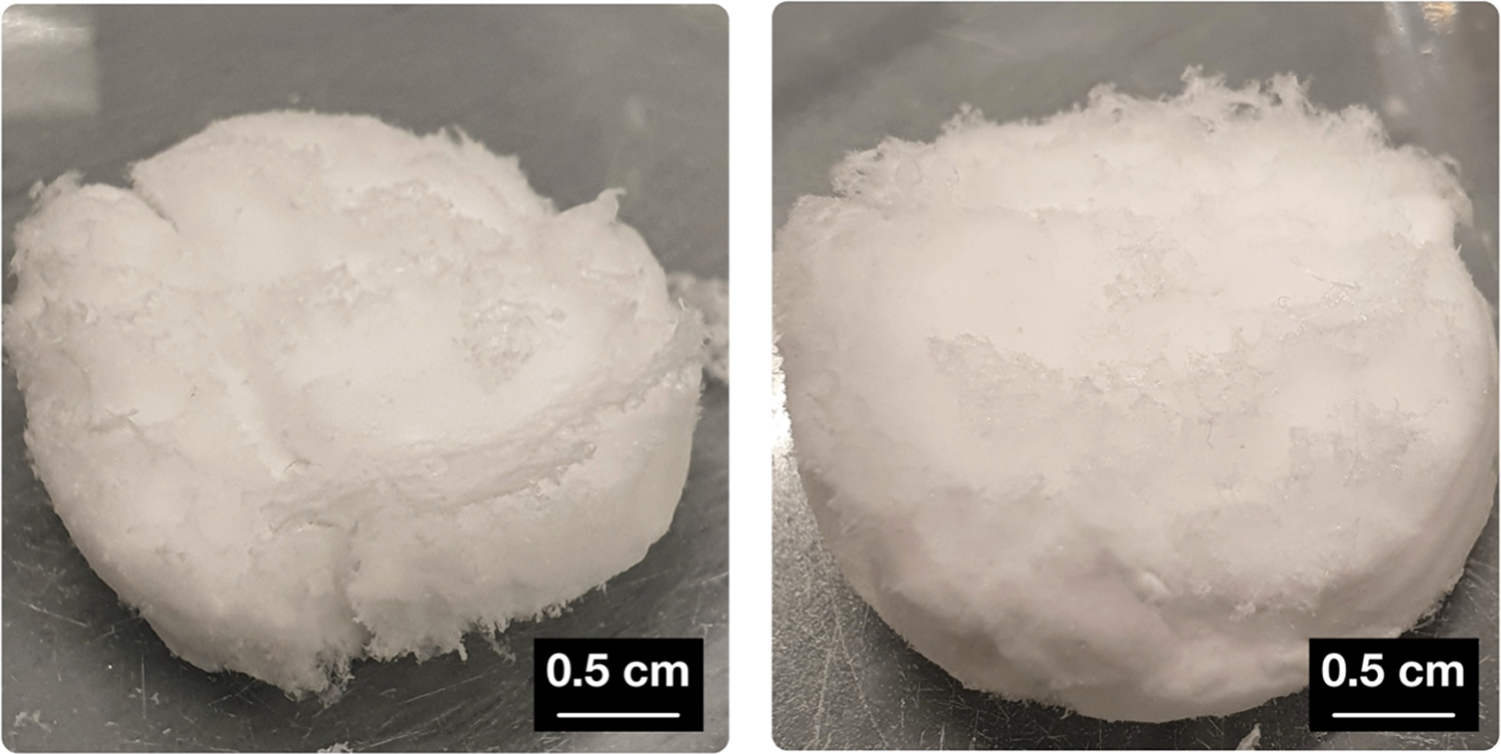
Photographs of pseudo-fibrin aerogels.

The fibrous hydrogels can easily be extracted from the surrounding medium and lyophilized. By this, pseudo-fibrin aerogels are accessible, as shown in Figure 2. The gained material is extremely light weight and appears very similar to cotton wool in haptics, flexibility, and stability. Moreover, the fibrous nature of the material becomes apparent already at optical evaluation. The depicted sample weighs just 73 mg at a volume of ≈3.4 cm^3^, resulting in a density of 0.021 g/cm^3^. From the bulk density of amorphous fibrinogen (0.13 g/cm^3^), the pore volume of this sample was estimated to be at least 85%. Video 2, showing the behavior and appearance of the aerogel, is again found in the Supporting Information.

**Figure 3:**
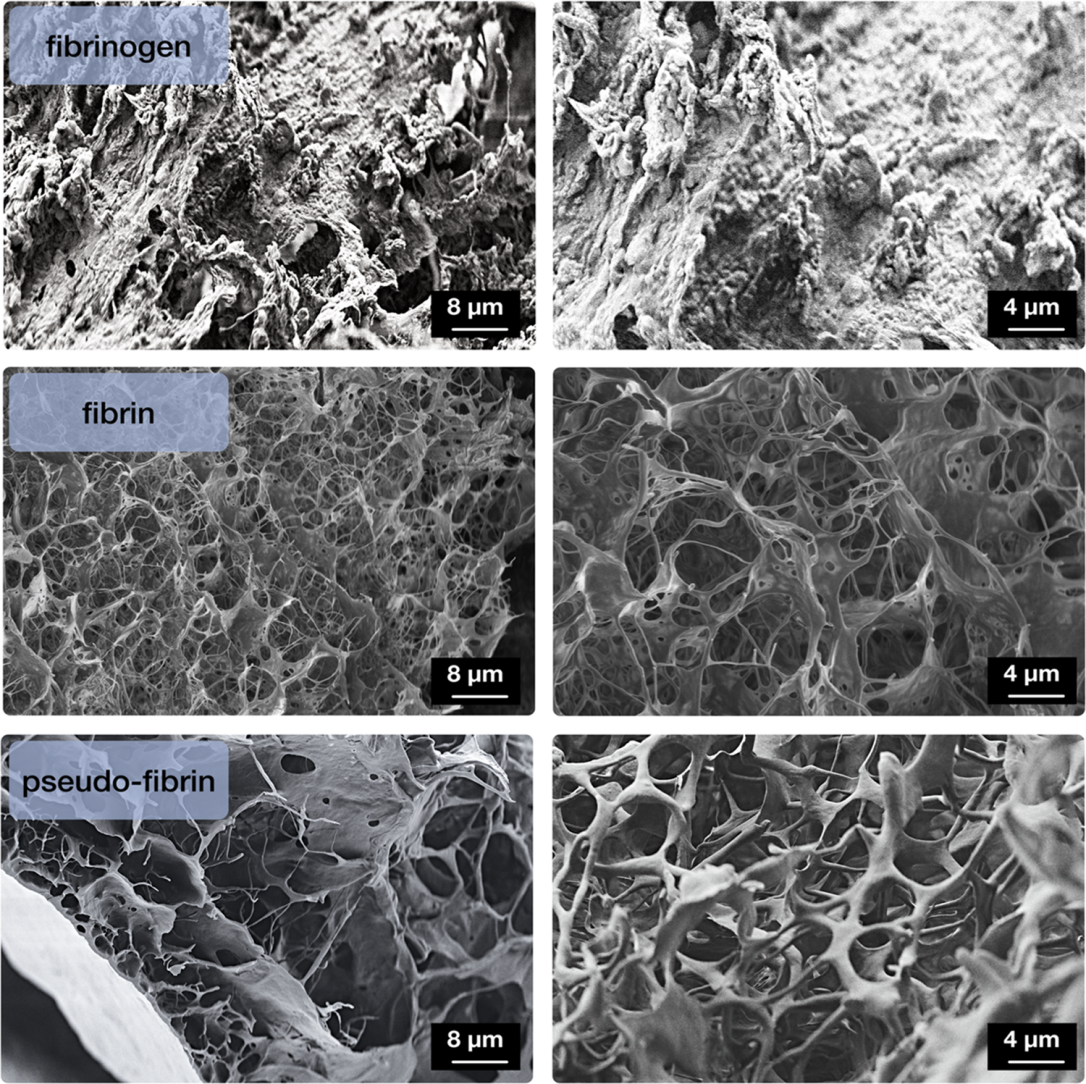
Structural comparison between fibrinogen, fibrin, and fibrinogen aggregates induced by phosphate ions (pseudo-fibrin) via SEM pictures.

The microscopy images from respective aerogel samples in Figure 3 finally reveal a highly fascinating microstructure. It consists of highly porous, fiber network structures. The pores appear to be interconnected and the protein structures are very thin. Moreover, Figure 3 also shows a comparison with the likewise lyophilized structures of native fibrinogen and enzymatically cross-linked fibrin. As expected, fibrinogen shows an amorphous structure if lyophilized from either dissolution or an unspecific aggregation by addition of pure water. Lyophilized fibrin again shows a porous structure with high similarity to pseudo-fibrin. Some more impressions on samples of pseudo-fibrin can be found in the Supporting Information (Figure S1).

The striking analogy of pseudo-fibrin to actual fibrin, both as hydro- and aerogel, renders the new material a promising alternative for fibrin in biomaterial applications. A much faster initiation of fiber formation, exclusion of possible thrombin residuals, and low-cost reagents are great advantages in comparison to fibrin. Regardless of such technical considerations, the observed phenomenon also represents the first large-scale, enzyme-free process of fibrillogenesis from fibrinogen in solution.

### 3.2 Optimization of Aggregation Conditions

It is important to state at this point, that the observed specific aggregation of fibrinogen into fibrils and finally hydrogel-like structures, strongly depends on very strict experimental conditions. To identify all relevant factors of the driving force for specific aggregation, qualitatively and quantitatively, several conditions of the process need to be discussed.

First, it was found that the process efficiency is profoundly enhanced by first dissolving fibrinogen in ultrapure water and adding the specific amount of phosphate buffer afterward. Another key to obtain the delicate, fibrous hydrogels is to avoid convection during the self-assembly, which means that the reaction mixture must not be stirred. Otherwise, all emerging fibers aggregate and form an unspecific precipitate.

**Figure 4:**
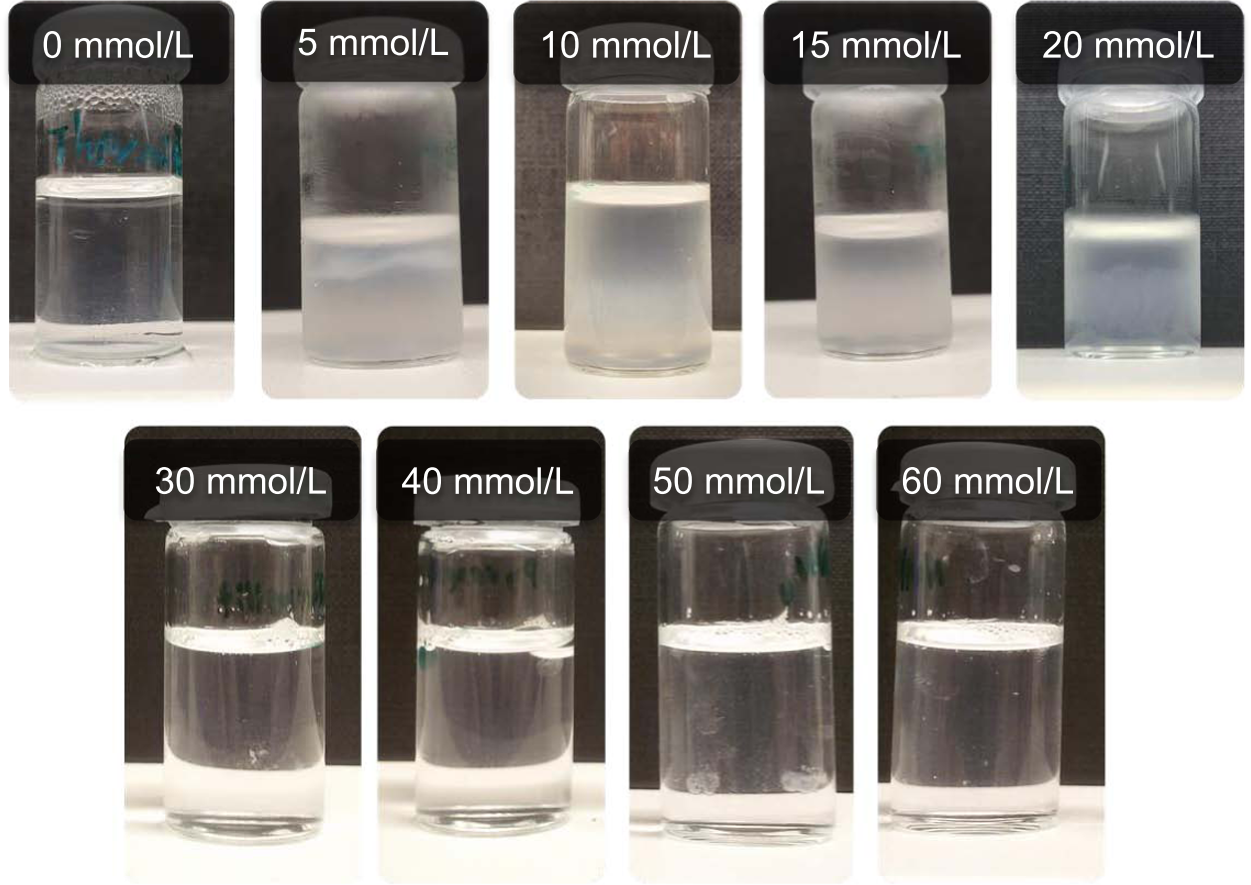
Appearance of fibrinogen solutions, 4 h after addition of varying phosphate concentrations. An optimum value for aggregation is observed at 15 mmol/L. All samples are at a constant pH of 7.0.

The most relevant aspect is the addition of a defined concentration of trigger salt, in particular (at this point) phosphate(s). If this narrow window is exceeded in either direction, the protein remains in solution. Phosphate concentrations between 5 and 20 mmol/L, with an optimum of 15 mmol/L, show particularly intense fiber formation after just a few seconds. Applied phosphate concentrations above 20 mmol/L significantly decrease yield and kinetics of fiber formation. This effect is even more pronounced when using phosphate concentrations above 60 mmol/L, where the fibrinogen remains completely dissolute (Figure 4).

Although this observation is not understood in full detail yet, some insight can be gained from our previous work, which focused on fibrinogen self-assembly triggered by lowering of ionic strength.^13^ The most plausible explanation at this time is therefore the consideration of two competing effects: At low salt concentrations, the amount of phosphate(s) is simply not sufficient to induce intense fiber formation. Instead, only low amounts of rather thin fibers are observed. Kinetics appears to be dependent on salt concentration at this level. At too high salt concentrations however, the dissolving effect, connected to an increased ionic strength, outweighs the specific aggregation effect and inhibits fiber formation.

Besides the balanced phosphate concentration, the amount of other salts, especially NaCl, is another important aspect to achieve fiber formation. As NaCl does not trigger the fiber formation but reduces aggregation with increasing ionic strength, its concentration should be as low as possible. At higher amounts, it inhibits fiber formation, similar to too high phosphate concentrations. Precisely, the concentration of NaCl should be below 20 mmol/L to gain significant fiber formation. From this point on, yield and kinetics decrease until, at a concentration of 140 mmol/L, the aggregation is completely inhibited. Once again, this effect is most likely connected to an increased ionic strength.

**Figure 5:**
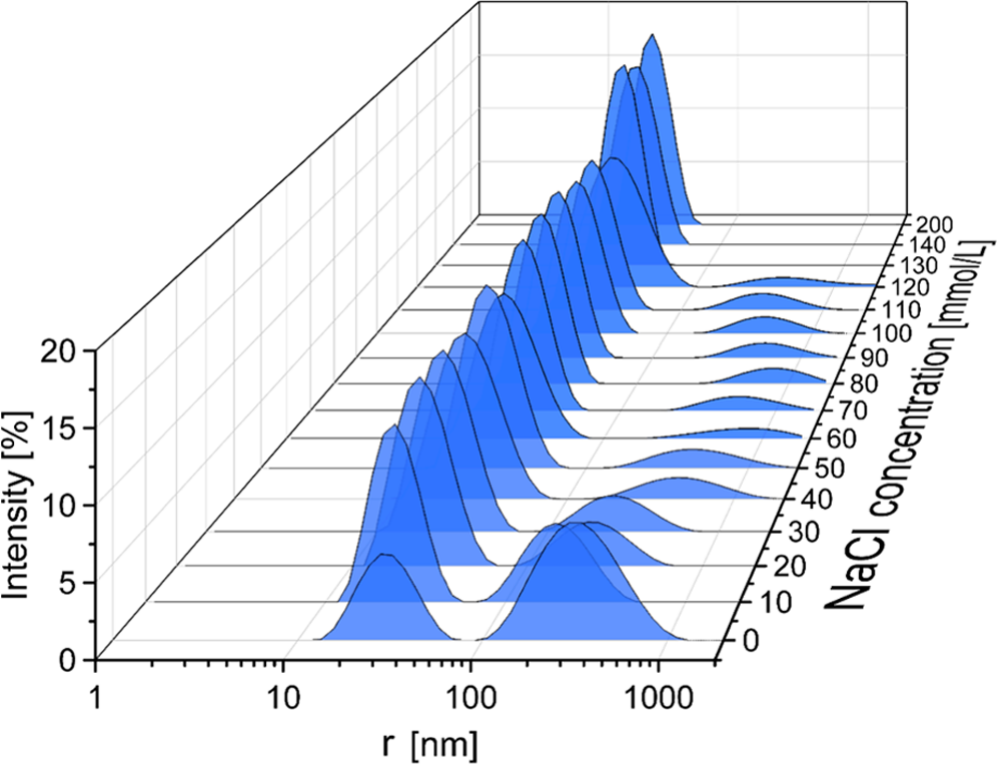
Size distribution of fibrinogen in solution at various concentrations of NaCl, gained via dynamic light scattering.

It should not be neglected in this context that fibrinogen is not completely dissolved on the molecular level at NaCl concentrations suitable for fiber formation. The size distributions in Figure 5 show this effect. Only at NaCl concentrations above 130 mmol/L, pure molecular fibrinogen is detected. With decreasing concentration of NaCl, an aggregate structure in the range of 300–400 nm becomes more and more prominent. It should be noted though, that the size distribution from dynamic light scattering is based on a weighting by intensities, which strongly favors larger particles. The actual number of aggregates is therefore much lower as it appears from the spectra. Nevertheless, it cannot be excluded that these aggregates play a role in the observed fiber formation, e.g., in the form of a seed for aggregation. It is at least a curious coincidence that the inhibition effect of NaCl correlates with the amount of aggregates.

Although the precise consideration of ion concentrations is the prime factor for a successful material buildup, the process can also be enhanced by tuning of additional conditions, especially pH and temperature.

**Figure 6:**
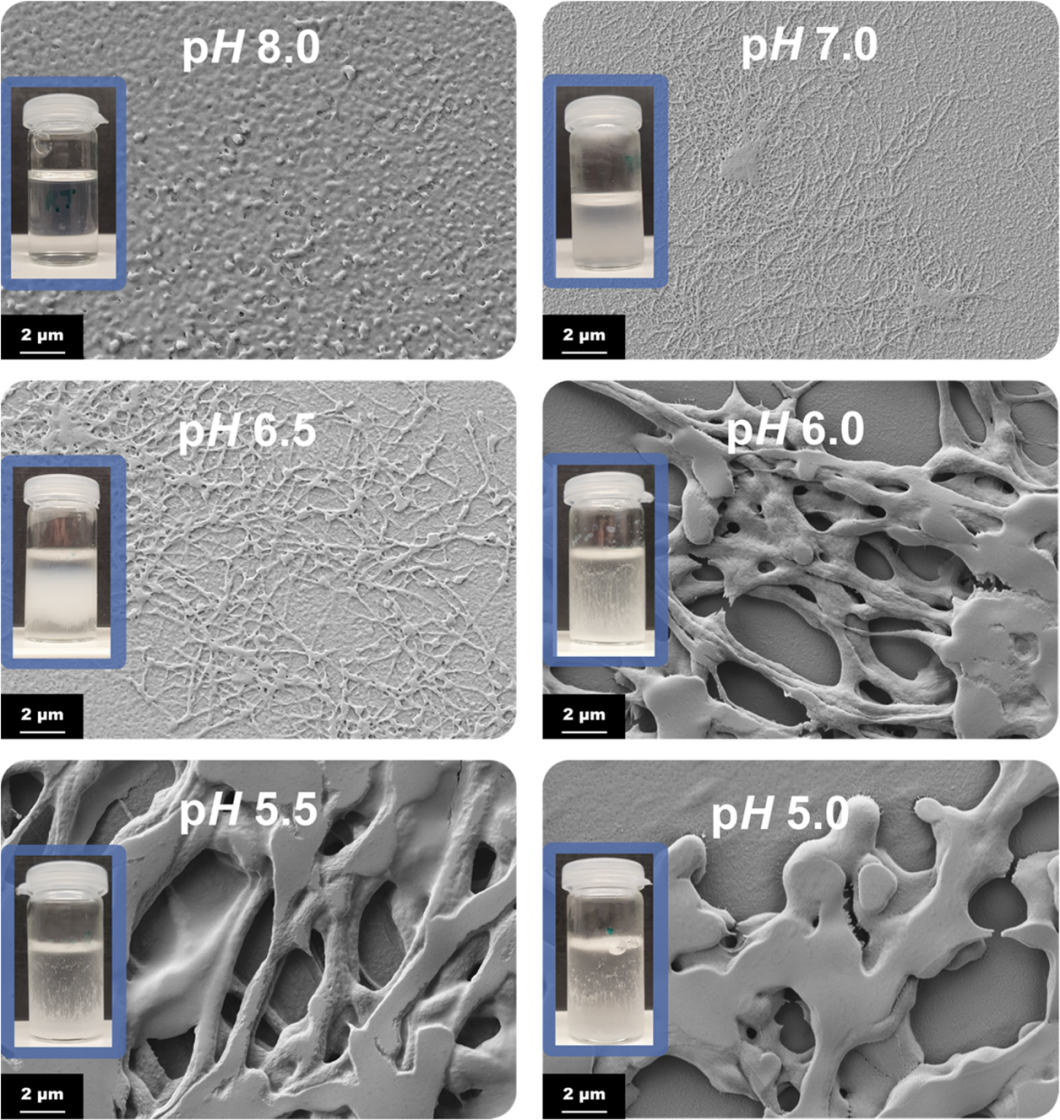
pH dependence of the pseudo-fibrin formation after 4 h reaction time. Best results are obtained between pH 7.0 and 6.5.

The most relevant parameter to be considered here is the pH of the reaction medium, as even small deviations lead to a drastically different aggregation behavior. Fiber formation is most successfully triggered at pH values between 6.5 and 7.5 as can be seen in Figure 6. Under more basic conditions, no precipitation occurs, and no fibrous material is observed after drying. More acidic pH conditions, on the other hand, lead to very rapid and massive precipitation. Microscopy images still show slight characteristics of fibrous material; however, fibers are extremely thick and highly aggregated. Also, the characteristic hydrogel-like structure is not obtained.

From these experiments, it becomes apparent that conditions closer to fibrinogen’s solubility limit, in this case, its isoelectric point of 5.5,^10^ promotes fibrillogenesis. If the pH is too close though, fibrillogenesis seems to be competing with amorphous precipitation. If it is too high, no fiber formation occurs. Furthermore, it should be noted at this point, that a pH shift alone does never induce fiber formation. Only in combination with the right amount of phosphate salt, fibrillogenesis occurs.

Following the concept of a necessary low solubility, as found for the pH conditions, the temperature of the experiments is considered next. Fibrinogen is commonly dissolved at 37 °C, again to closely mimic biological conditions. However, the described self-assembly process is not triggered at all at this temperature. At 20 °C however, fiber formation becomes slightly visible. And further reduction of the temperature to values as low as 5 °C drastically improves yield and kinetics of fibrillogenesis. At this temperature, phosphate addition leads to an instantaneous and much denser cloud formation. Even lower temperatures are however counterproductive for the process, because now fibrinogen solubility is strongly limited. The necessary concentration to gain stable hydrogel-like structures at the end of the process has been determined to be 5 g/L, which cannot be achieved at temperatures below 5 °C. At lower concentrations, fiber formation still occurs but in lower quantities which are apparently insufficient to form the dense hydrogel-like material.

All results from the described experiments confirm the hypothesis that the fibrous aggregation is most effective at conditions close to fibrinogen’s solubility limit, i.e., low temperatures, a low ion concentration (to avoid salting-in effects) and a pH near the isoelectric point. At such conditions, presence of the right amount of phosphate ions induces the controlled, fibrous aggregation and eventually the formation of pseudo-fibrin gels.

### 3.3 Specification and Variation of Aggregation Trigger

The question remains at this point, whether the fibrous aggregation is an effect specific to phosphate or if it can be achieved with other reactants as well. A prospective starting point for this investigation is the utilization of anions with similar features to phosphate. For this purpose, sodium citrate was first tested as a reactant because it also contains three negative charges located at oxygen atoms. And indeed, citrate was found to trigger the same fibrous self-assembly process, proving that the reaction is not limited to or a specific effect of phosphate.

A closer look at the two salts, however, bears another complication. In pure form, both phosphate and citrate inherit three negative charges. Actually, as consideration of p*K*_a_ values (Table 1) reveals, only citrate has three negative charges at pH 7.0. Phosphate, on the contrary, rather exists in an equilibrium of hydrogen- and dihydrogen phosphate at this pH, and thus mostly carries no more than two charges. Although a small portion of the equilibrium might be at three charges for phosphate as well, presence of three negative charges as the main prerequisite for the function as trigger appears at least doubtful.

**Table 1:** p*K*_a_ Values of H_3_PO_4_ and Citric Acid and the Charge of the Dominant Species at pH 7.0

	p*K*_a_^21^	dominant species at pH 7.0
H_3_PO_4_	2.2/7.2/12.3	H_2_PO_4_^–^
C_6_H_8_O_7_	3.1/4.8/6.4	C_6_H_5_O_7_^3–^

Another mutuality of phosphate and citrate is that their charges are located at oxygen atoms. And indeed, Prussian red (K_3_[Fe(CN)_6_]), an anion with likewise three charges but without oxygen, failed to induce any fiber formation at all. Another control experiment was performed with 2-ethyl-2-(hydroxymethyl)propane-1,3-diol (TMP), also known as 1,1,1-trimethylolpropane, as an example of a neutral but oxygen-containing molecule. Again however, no fiber formation was observed. This leads to the conclusion that all suitable triggers so far are oxygen-containing acid anions.

To further investigate this hypothesis, several other acid anions were examined (Table 2). From this, it was found that fibrillogenesis can, besides phosphate, be induced especially by sodium salts of sulfate, tartaric acid, and citric acid. In addition, sodium salts of nitrate, acetate and pyromellitic acid (1,2,4,5-benzentetracarboxylic acid) induce a, however weak, fiber formation. All successful experiments show similar fibrous structures in strong contrast to standard fibrinogen. On the other side of the line, chloride, iodide, and thiocyanate induced no fiber formation at all.

**Table 2:** Reactants Utilized and Their Effect on Pseudo-Fibrin Formation^a^

reactant	no. of charges at pH = 7.0	charge(s) located at oxygen atom	fiber formation
C_6_H_14_O_3_ (TMP)	0	no	none
NaCl	1	no	none
NaI	1	no	none
NaSCN	1	no	none
NaNO_3_	1	yes	very weak
NaCH_3_COO (acetate)	1	yes	weak
Na_2_SO_4_	2	yes	strong
Na_2_C_4_H_4_O_6_ (tartrate)	2	yes	strong
NaH_2_PO_4_/Na_2_HPO_4_	1–2	yes	strong
Na_3_C_6_H_5_O_7_ (citrate)	2–3	yes	strong
K_3_[Fe(CN)_6_]	3	no	none
Na_3_C_10_H_3_O_8_ (pyromellitic acid)	4	yes	weak

a Effective ions have in common charges located at oxygen atoms.

To shine more light on the nature of suitable triggers, the kosmotropic effect of the ions, as found in the Hofmeister series, was also considered. Since there is no “universal” Hofmeister series found in the literature, rather slightly different ones, Table 3 orders the investigated anions in a general way from kosmotropic to chaotropic.^21−24^

**Table 3:** Anions Utilized and Their Approximate Position in the Hofmeister Series

strong kosmotropic effect	weak kosmotropic effect	weak chaotropic effect	strong chaotropic effect
SO_4_^2–^	CH_3_COO^–^ (acetate)	Cl^–^	I^–^
C_4_H_4_O_6_^2–^ (tartrate)			
C_6_H_5_O_7_^3–^ (citrate)		NO_3_^–^	SCN^–^
H_2_PO_4_^–^/HPO_4_^2–^			

From Table 3, it becomes obvious that all currently suitable anions for pseudo-fibrin formation are kosmotropic. If the anion becomes more chaotropic, much less and rather thin fibers are obtained (e.g., with acetate). No fiber formation—not even at low pH—was observed for the listed non-oxygen-containing anions, which often exhibit a strong chaotropic effect. At this point, it cannot be distinguished whether kosmotropy or the presence of (multiple) charged oxygen atoms or a combination thereof are the driving force behind the fibrous aggregation since these conditions often occur together. Nevertheless, it has been shown that the fiber formation is not some kind of phosphate-specific effect but is rather related to specific ionic species. This opens up possibilities for a wide range of triggers to choose from for different occasions.

### 3.4 Rheological Properties of Pseudo-Fibrin

**Figure 7:**
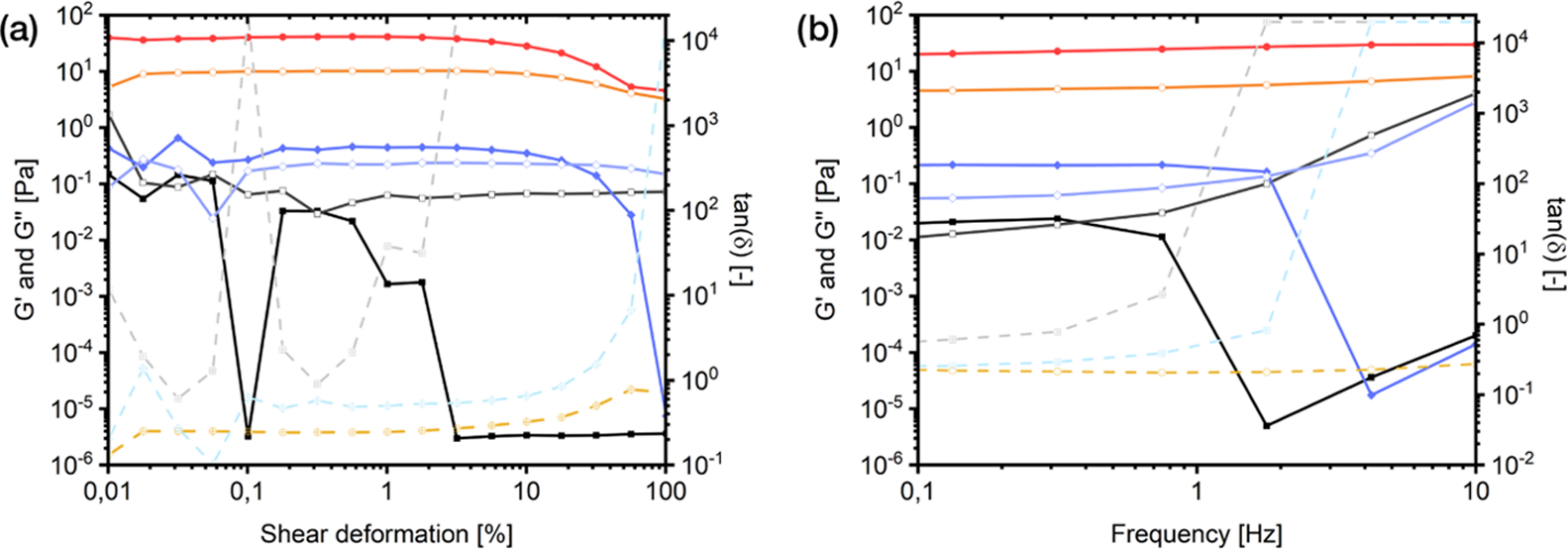
Rheological characterization of fibrinogen (), fibrin (red circle solid), and pseudo-fibrin (blue diamond solid) by means of an amplitude sweep (a) and a frequency sweep (b). Filled symbols = *G*′; hollow symbols = *G*″; crossed symbols/dashed lines = tan (δ).

To initially characterize the viscoelastic properties of pseudo-fibrin, its storage and loss modulus were measured by means of an amplitude sweep and a frequency sweep, respectively. The results are compared to unstructured fibrinogen and enzymatically produced fibrin (Figure 7). It should be noted that only a qualitative comparison can be achieved at the current state of research. A more profound mechanical characterization will be presented in a future complementary study.

First, the linear viscoelastic region of all variants was determined by an amplitude sweep. It is seen that pseudo-fibrin’s properties are qualitatively comparable to that of fibrin. Both materials actually show gel-like characteristics since the storage modulus *G*′ is higher than the loss modules *G*″ and both values run mostly parallel. This is also confirmed by the values of tan (δ) in the linear viscoelastic region. However, the quantitative values show that fibrin is significantly more stable, which is also observed in the handling of the materials.

On the other hand, there is a significant difference from pseudo-fibrin to its precursor fibrinogen, which shows a more erratic behavior, much lower absolute moduli, and a significantly earlier intersection at deformations around 3%. The behavior of fibrinogen is therefore more comparable to unstructured aggregates, which are easily disintegrated. Pseudo-fibrin however is stable until a deformation of 20%, resembling a more gel-like behavior, similar to fibrin. At this point, we again remind that the only difference between fibrinogen and pseudo-fibrin is the addition of 15 mmol/L of phosphate, leading to the described changes in rheological behavior.

Additional measurements were performed with variation of the frequency. For this, a constant shear deformation of 1% was applied since all variants show linear viscoelastic behavior at this value. Once again, the overall rheological behavior of fibrinogen significantly changes toward that of fibrin after addition of phosphate. Especially the point of intersection between *G*′ and *G*″ shifts to higher frequencies for pseudo-fibrin (2 Hz), meaning higher stability compared to native fibrinogen (0.75 Hz).

In comparison with literature data, the measured absolute moduli of fibrin are still comparatively low. While in the literature, values of *G*′ between 1000 and 10 000 Pa are found,^25^ we only measured values from 20 to 30 Pa. This difference is most likely due to the unusually low concentration of 5 g/L fibrinogen, resulting in a lower stability. Another factor constitutes the unusual conditions with low temperatures and nonbiological salt environments. To accommodate, fibrin and fibrinogen have also been investigated at room temperature in PBS buffer, which is sketched in the Supporting Information (Figure S2). Due to the inherent requirements, pseudo-fibrin can of course not be obtained under these conditions. It is observed that these conditions play a minor role in the qualitative results, as higher absolute values for *G*′ and *G*″ are seen. However, concentration effects seem to be the dominant factor.

In conclusion, pseudo-fibrin can significantly be distinguished from native fibrinogen. Although it does not reach the stability of actual fibrin yet, gel-like behavior is clearly observed. As this is just an initial report, the properties of pseudo-fibrin can most certainly be increased, e.g., by adding an additional cross-linker such as glutaraldehyde after the successful fibrillogenesis. Improvement of the mechanical properties in general is a major subject of our ongoing study into pseudo-fibrin.

### 3.5 Investigation of Initial Stage of Aggregation

**Figure 8:**
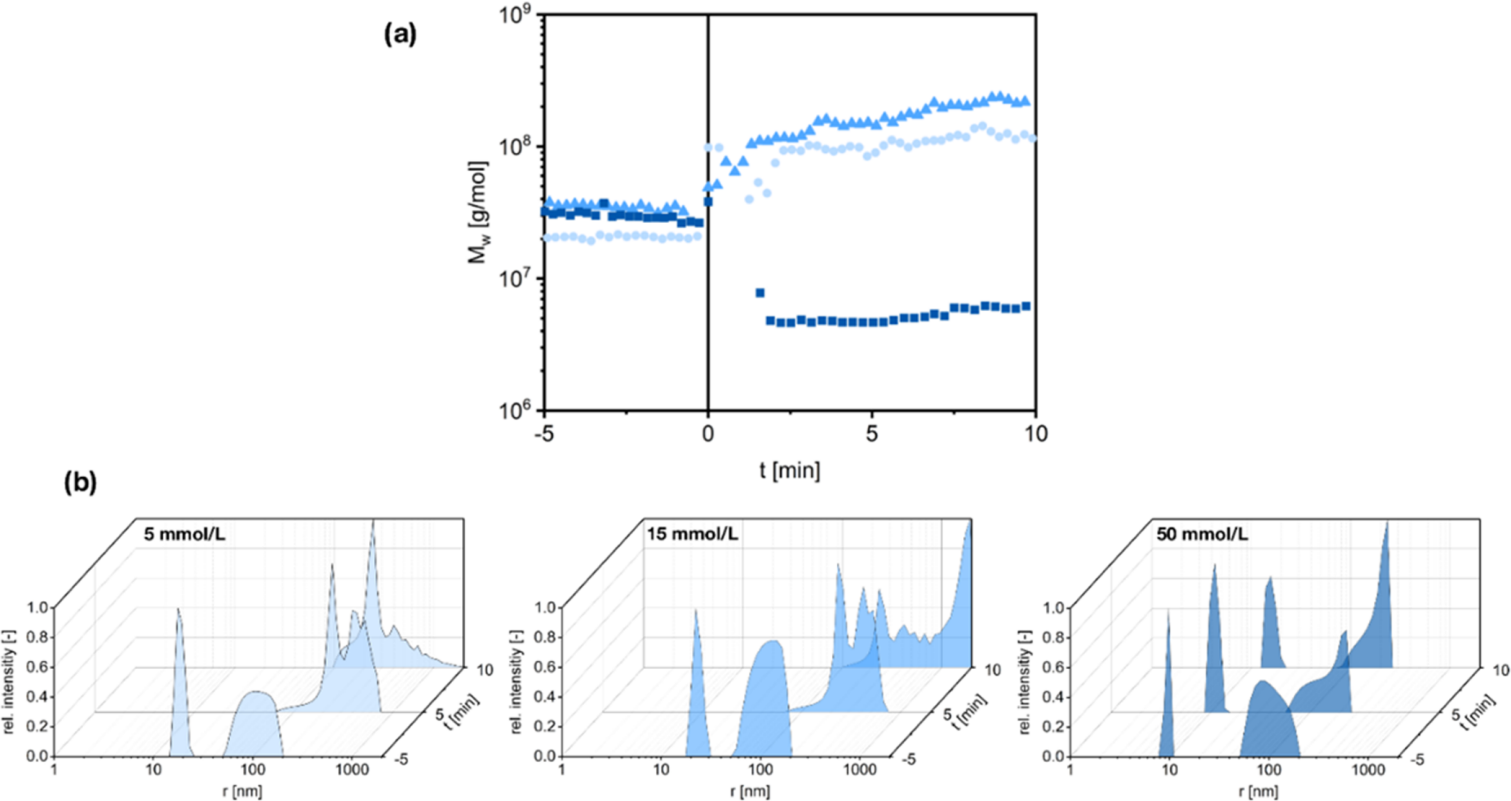
Results of time-resolved SLS/DLS measurements; *t* = 0 corresponds to the initiation by adding respective amounts of phosphate buffer. (a) Apparent molar mass values over time for (blue circle solid) 5 mmol/L, (blue triangle up solid)15 mmol/L, and (blue box solid) 50 mmol/L phosphate buffer. (b) Size distributions from DLS before and after initiation for various concentrations of phosphate buffer, recorded at a scattering angle of 86°.

To get a more profound insight, the initial stage of fibrous aggregation was also examined via time-resolved static and dynamic light scattering (SLS/DLS), depicted in Figure 8. In these experiments, addition of phosphate buffer is defined as *t* = 0. Evolution of the molar aggregate mass indicates that aggregation is initiated immediately after addition of 5 or 15 mmol/L phosphate buffer, respectively. Moreover, the final molecular mass is higher at 15 mmol/L, which supports the previously stated hypothesis of two competing effects. On the other hand, addition of too high amounts (represented by 50 mmol/L), does not trigger significant aggregation at all. The molar mass even decreases, possibly because the small aggregates, present in the base solution (see Figure 5), disintegrate. As the fibrous aggregates quickly become too large for the utilized setup, further information on the nature of aggregation is hard to determine from those time-resolved light scattering measurements. However, the behavior is dissimilar from that described in our previous work.^13^ Therefore, it is at least no contradiction to the proposed fibrillogenesis.

Comparison of size distributions from DLS gives further insight into the aggregation behavior. For *t* < 0, the expected pattern, as described before is clearly visible in all measurements. For *t* > 0, however, a clear differentiation between the various concentrations can be seen. At 50 mmol/L, only very slight aggregation is observed, while the peak for molecular fibrinogen is retained. Once again it should be mentioned that the intensity of DLS signals strongly favors larger particles. For lower phosphate concentrations, molecular fibrinogen disappears entirely. Instead, a strong aggregation is detected. Moreover, this aggregation is clearly stronger in the case of 15 mmol/L, especially seen after 10 min. Here, much larger particles, which are already way out of range of the light scattering setup, are observed. These results fit very well to the macroscopically observed clouding effect, which is strongest at 15 mmol/L phosphate.

### 3.6 Integrity of Protein Structures

**Figure 9:**
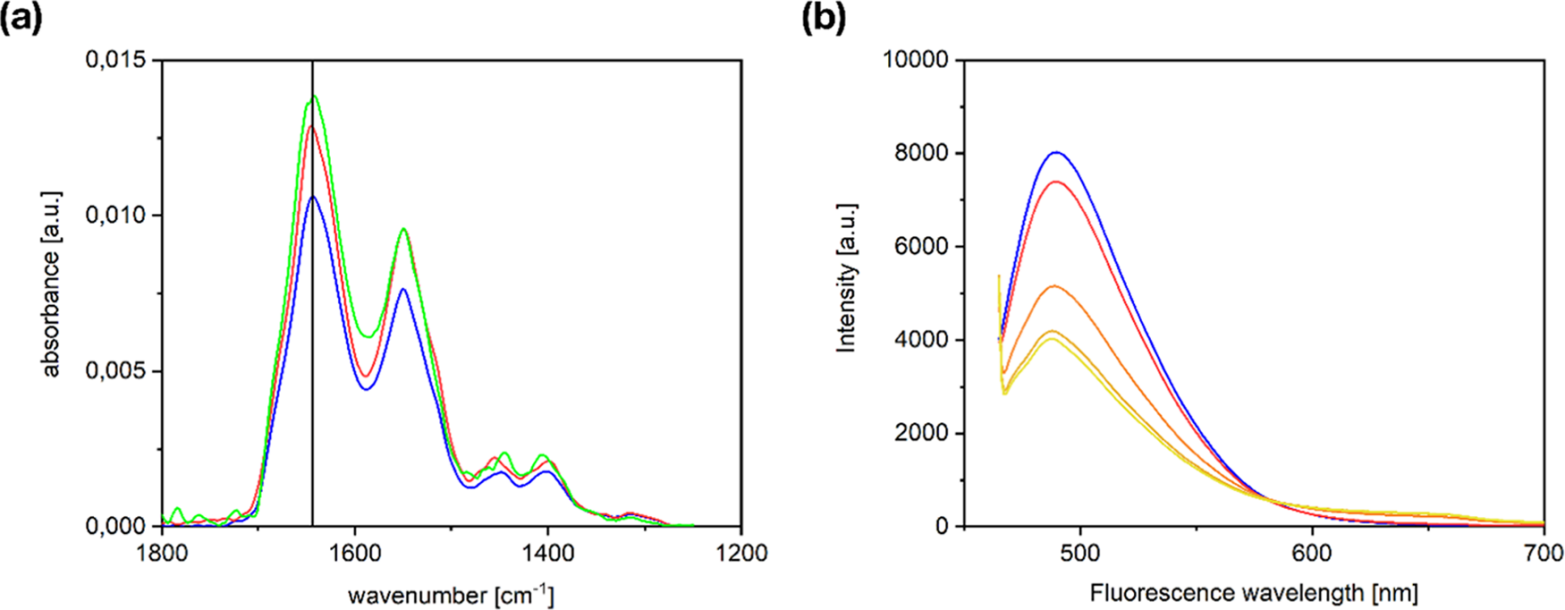
(a) ATR-IR spectra of unstructured fibrinogen (blue circle solid), fibrin (red circle solid), and pseudo-fibrin created with phosphate (green circle solid). All spectra are identical regarding especially the amide I bands, which are located at the same wavenumber (marked with a black line). Consequently, the three-dimensional structure of fibrinogen is the same in all three cases and unwanted amyloidosis can be excluded as a reason for pseudo-fibrin formation. (b) Fluorescence intensities of (blue circle solid) a ThT-containing fibrinogen solution and pseudo-fibrin (red circle solid) 10 s, (orange circle solid) 5 min, (gold circle solid) 10 min, and (yellow circle solid) 15 min after phosphate addition.

A final question that shall be considered here is whether the fibrous aggregation might be due to conformational changes in the protein itself. Ensuring the integrity of the native state is of high significance for potential applications of pseudo-fibrin as a biomaterial. The closer the natural state is retained, the better the behavior in a biological environment can be predicted. One aspect which needs especially to be ruled out is the formation of amyloid fibers (β-sheet aggregates) as cause for the fibrous arrangement of pseudo-fibrin, as they are known to be harmful.^26,27^ To exclude such unwanted amyloidosis, attenuated total reflection infrared (ATR-IR) and fluorescence spectra were recorded for native fibrinogen, enzymatically triggered fibrin, and samples of pseudo-fibrin (Figure 9).

The resulting IR spectra in Figure 9a clearly show the amide I (1650 cm^–1^), II (1550 cm^–1^), and III (1400–1200 cm^–1^) bands, which contain information about the three-dimensional structure of a protein.^28^ As all three spectra look practically identical, fibrinogen, fibrin, and pseudo-fibrin inherit at least a very similar three-dimensional folding. Small deviations of the spectra can be neglected due to the overall low absorbance. Most importantly, the results confirm that no amyloid formation occurs and the self-assembly to pseudo-fibrin retains the native state of the protein.

Additional fluorescence investigations using a thioflavin T (ThT) binding assay led to the same conclusion. In the presence of ThT, β-sheet structures show intense fluorescence at 482 nm when excited at a wavelength of 450 nm.^29^ Consequently, β-sheet-rich amyloids would lead to a strong increase in fluorescence intensity. Figure 9b compares the fluorescence spectra of native fibrinogen in solution and pseudo-fibrin for various times after phosphate addition. The basic fluorescence of the reference indicates the native β-sheet amount within fibrinogen. With increasing time after phosphate addition, the fluorescence intensity does not increase but rather decreases, due to the strong scattering produced by the protein cloud. An amyloidosis induced by phosphate ions would however overcompensate this effect by far. A significant increase in fluorescence intensity would be observed. Since this is not the case, unwanted amyloidosis can once again be ruled out.

## 4 Conclusions

A groundbreaking and nevertheless highly feasible technique enables fibrillogenesis and subsequent gelation of fibrinogen via salt-induced self-assembly. Highly porous and flexible hydrogels as well as aerogels can be obtained by adding oxygen-containing, preferably multivalent acid anions like phosphate or citrate to a fibrinogen solution in defined environments. Optimum conditions for fibrillogenesis are found on the threshold of fibrinogen’s solubility limit, i.e., a slightly acidic pH, temperatures around 5 °C and absence of NaCl or otherwise increased ionic strength. Potential thrombin residuals could be ruled out as trigger for fibrillogenesis as well as unwanted amyloidosis. The mechanical stability of pseudo-fibrin is still lower than that of fibrin. However, its rheological behavior can significantly be distinguished from native fibrinogen and shows profound gel-like characteristics.

The 3D structures and the behavior of the produced gels show striking analogies of the investigated pseudo-fibrin to actual fibrin. Pseudo-fibrin therefore retains the advantages but overcomes the problems of fibrin in biomaterial applications. A much faster initiation of fiber formation, exclusion of possible thrombin residuals, and low-cost reagents are the main benefits. Regardless of such technical considerations, the observed phenomenon also represents the first enzyme-free process of large-scale fibrillogenesis from fibrinogen in solution.

Of course, these findings are just the beginning and open up a huge range of future research possibilities, especially toward better stability and the understanding of the fiber formation mechanism. Already at this initial stage, however, the material shows great potential toward an all-purpose, enzyme-free fibrillogenesis.

## Supporting Information Available

SEM impressions on the structure of pseudo-fibrin in aerogel form and additional rheological characterization of fibrin and fibrinogen (PDF)
Production of pseudo-fibrin (Video 1) (AVI)
Appearance of pseudo-fibrin aerogel (Video 2) (AVI)

## Supplementary Material

bm1c00489_si_001.pdf

bm1c00489_si_002.avi

bm1c00489_si_003.avi
