# Supplementary material for: Self-Assembled Fibrinogen Hydro- and Aerogels with Fibrin-like 3D Structures
Source: Biomacromolecules. 2021 Aug 19;22(10):4084–94. doi: 10.1021/acs.biomac.1c00489 (PMC8512673; doi:10.1021/acs.biomac.1c00489)
Supplement: Supplementary file 1 — bm1c00489_si_001.pdf [file bm1c00489_si_001.pdf]

Supporting Information - Self-assembled fibrinogen hydro- and aerogels with fibrin-like 3D-structures

*Dominik Hense[1,2], Anne Büngeler[2], Fabian Kollmann[3], Marcel Hanke[4], Alejandro Orive[4], Adrian Keller[4], Guido Grundmeier[4], Klaus Huber[3], and Oliver I. Strube[1,2]**

[1] Institute for Chemical Engineering, University of Innsbruck, Austria

[2] Biobased and Bioinspired Materials, Paderborn University, Germany

[3] Physical Chemistry, Paderborn University, Germany

[4] Technical and Macromolecular Chemistry, Paderborn University, Germany

* corresponding author: oliver.strube@uibk.ac.at, phone: +43 512 50755300

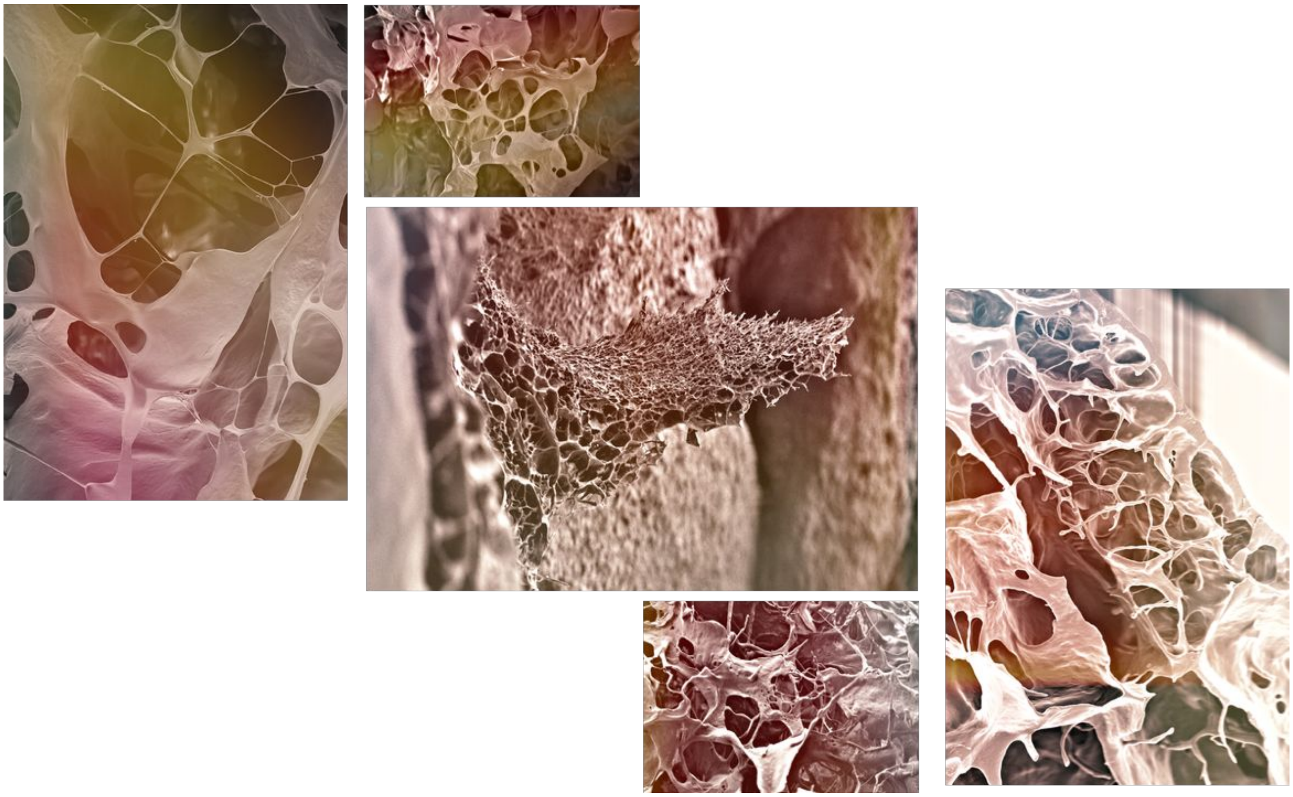

Figure S1. Impressions on the structure of pseudo-fibrin in aerogel form. The filigree and highly porous structure of the material becomes apparent.

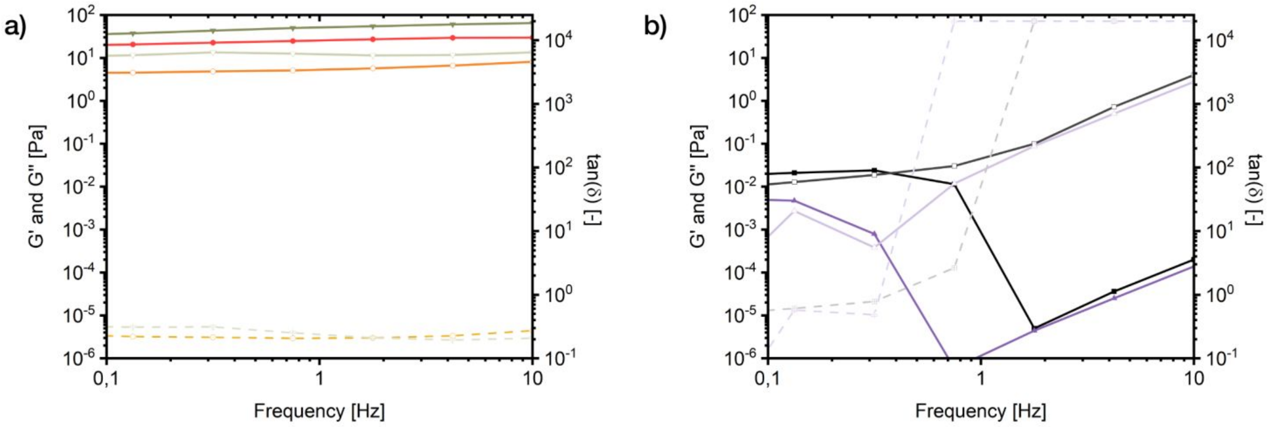

Figure S2. Rheological characterization of (a) fibrin and (b) fibrinogen at room temperature under standard conditions, i.e. 5 °C, salt free (●/□) and in PBS buffer at room temperature (▼/▲). Filled symbols = G'; hollow symbols = G''; crossed symbols / dashed lines = tan (δ).

Video 1. Pseudo-fibrin hydrogel: This video shows the initiation of fiber formation (slow motion), the gelation process towards the hydrogel (time lapse), and an impression of the behavior of the hydrogel (real time).

Video 2. Pseudo-fibrin aerogel: This video gives an impression of the behavior of the lyophilized aerogels regarding their fibrous nature, flexibility, and light weight.
